# *Staphylococcus cohnii* infection diagnosed by metagenomic next generation sequencing in a patient on hemodialysis with cirrhotic ascites: a case report

**DOI:** 10.3389/fcimb.2023.1240283

**Published:** 2023-09-22

**Authors:** Yu Lei, Qiaozhen Guo, Jingmei Liu, Huanjun Huang, Ping Han

**Affiliations:** Department of Gastroenterology, Tongji Hospital of Tongji Medical College, Huazhong University of Science and Technology, Wuhan, Hubei, China

**Keywords:** metagenomic next generation sequencing (mNGS), spontaneous bacterial peritonitis (SBP), *Staphylococcus cohnii* (*S.cohnii*), ascites, hemodialysis

## Abstract

**Background:**

Patients with spontaneous bacterial peritonitis (SBP) often just receive empirical antibiotic therapy, as pathogens can be identified in only few patients using the techniques of conventional culture. Metagenomic next generation sequencing (mNGS) is a useful tool for diagnosis of infectious diseases. However, clinical application of mNGS in diagnosis of infected ascites of cirrhotic patients is rarely reported.

**Case presentation:**

A 53-year-old male with cirrhosis on regular hemodialysis presented with continuous abdominal pain. After treatment with empiric antibiotics, his inflammatory parameters decreased without significant relief of abdominal pain. Finally, based on ascites mNGS detection, he was diagnosed as infection of *Staphylococcus cohnii* (*S.cohnii*), a gram-positive opportunistic pathogen. With targeted antibiotic treatment, the bacterial peritonitis was greatly improved and the patient’s abdominal pain was significantly alleviated.

**Conclusions:**

When conventional laboratory diagnostic methods and empirical antibiotic therapy fail, proper application of mNGS can help identify pathogens and significantly improve prognosis and patients’ symptoms.

## 1 Introduction

Bacterial peritoneal infection remains the most common triggering event for hospitalization of patients with cirrhosis (Aithal et al., 2021). Spontaneous bacterial peritonitis (SBP) is a severe and often fatal complication in patients with cirrhosis and ascites (European Association for the Study of the L, 2010). Although the pathogenesis of SBP is not fully understood, it is generally believed to be closely related to the factors listed below. These includes the diminished anti-infective immune defense caused by intestinal wall bruising and edema, the compromised mucosal barrier, impaired bacterial proliferation and bacterial translocation in the intestinal lumen, and the weakened monocyte-macrophage system (Wiest et al., 2012). Aerobic bacteria make up the majority of causing pathogens of SBP, and single-strain infections are the most common type. The majority of these pathogenic organisms come from the intestinal flora, with a small number from the urinary tract, respiratory symptom, and infected lesions of the soft tissues. Targeted anti-microbial therapy is critical for reducing mortality in SBP (Dever and Sheikh, 2015). However, as pathogens can be identified in a minority of patients with conventional culture techniques, most patients still receive empirical anti-microbial therapy (Li et al., 2022).

Metagenomic next generation sequencing (mNGS) is an emerging approach with the potential for pathogen screening (Miao et al., 2018). It can greatly increase diagnostic accuracy in the case of negative conventional culture methods (Chen et al., 2023). However, there are few clinical reports on the identification of SBP pathogens by mNGS. In this report, we present the first case of SBP infected by *Staphylococcus cohnii* (*S.cohnii*) diagnosed by mNGS of ascites, with a negative serology examination and conventional ascites culture. According to the mNGS result, the patient received efficient clinical antibiotic treatment and recovered.

## 2 Case presentation

A 53-year-old man was brought to the emergency department because of continuous abdominal pain and bloating for 3 days. He didn’t consume any fatty meals or alcohol before the commencement of the sickness, and both his farting and stools were normal. He has a 10-year history of chronic viral hepatitis B and a 6-year history of hypertension. He was diagnosed as chronic renal failure 7 years ago and had been receiving hemodialysis on a regular frequency (three times a week) through a left forearm arteriovenous fistula since 4 years ago. Moreover, he also underwent splenectomy due to splenomegaly and received endoscopic treatment for esophageal varices 2 years ago. He denied a history of other systemic diseases, allergies, or any relevant family history. Physical examination indicated tenderness and rebound pain in the mid and right abdomen, hyperactive bowel sounds on auscultation, and a positive percussion mobile turbid examination, while other physical examinations revealed no significant abnormalities. Laboratory testing showed a significant elevation of the blood potassium level (6.99 mmol/L, reference range =3.50-5.10 mmol/L), white blood cell count (14.26×10^9/L, reference range =3.5-9.5 ×10^9/L), and procalcitonin level (41.60 ng/mL, reference range = 0.02-0.05 ng/mL). The abdominal computed tomography (CT) scan showed liver cirrhosis, splenic postoperative changes, edema of the stomach wall and part of the colon wall, shrinkage of the left kidney, hydronephrosis and cortical thinning of the right kidney, and significant fluid accumulation in the abdominopelvic cavity. The patient was initially admitted to the department of nephrology due to hyperkalemia. He was treated with hemodialysis, and intravenous antibiotics (cefoperazone/tazobactam 2.25g per day and ornidazole 0.25g per 12h over 4 days, moxifloxacin 0.4g per day over 4 days), antispasmodic and analgesic therapy, among others. After the aforementioned medication were administrated, the levels of white blood cell count and blood potassium recovered to normal, and the levels of procalcitonin also showed a significant decrease. However, the patient’s abdomen pain was not well-controlled. There was no improvement in fluid in the abdominal cavity or edema in the intestinal wall a second CT scan, either.

For further diagnosis and treatment, the patient was then transferred to our gastroenterology department. Repeated laboratory investigation results showed a decrease of procalcitonin (down to 10.4ng/mL, reference range = 0.02-0.05 ng/mL) and a normal white blood cell count level (7.01×10^9/L, reference range =3.5-9.5×10^9/L). Depending on the clinical symptoms, laboratory, and imaging data after admission, the preliminary diagnosis of SBP was taken into consideration. Based on the diagnosis, empirical antibiotic therapy (biapenem 0.3g per 12h) was administered. He also received percutaneous paracentesis and drainage, and ascites specimens were sent for routine laboratory biochemical examination and cytology of body fluids, as well as bacterial culture. The number of nucleated cells in the ascites exceeded 19,000×10^6/L and the Rivalta test was remarkedly positive, while bacterial culture test results were negative.

**Figure 1:**
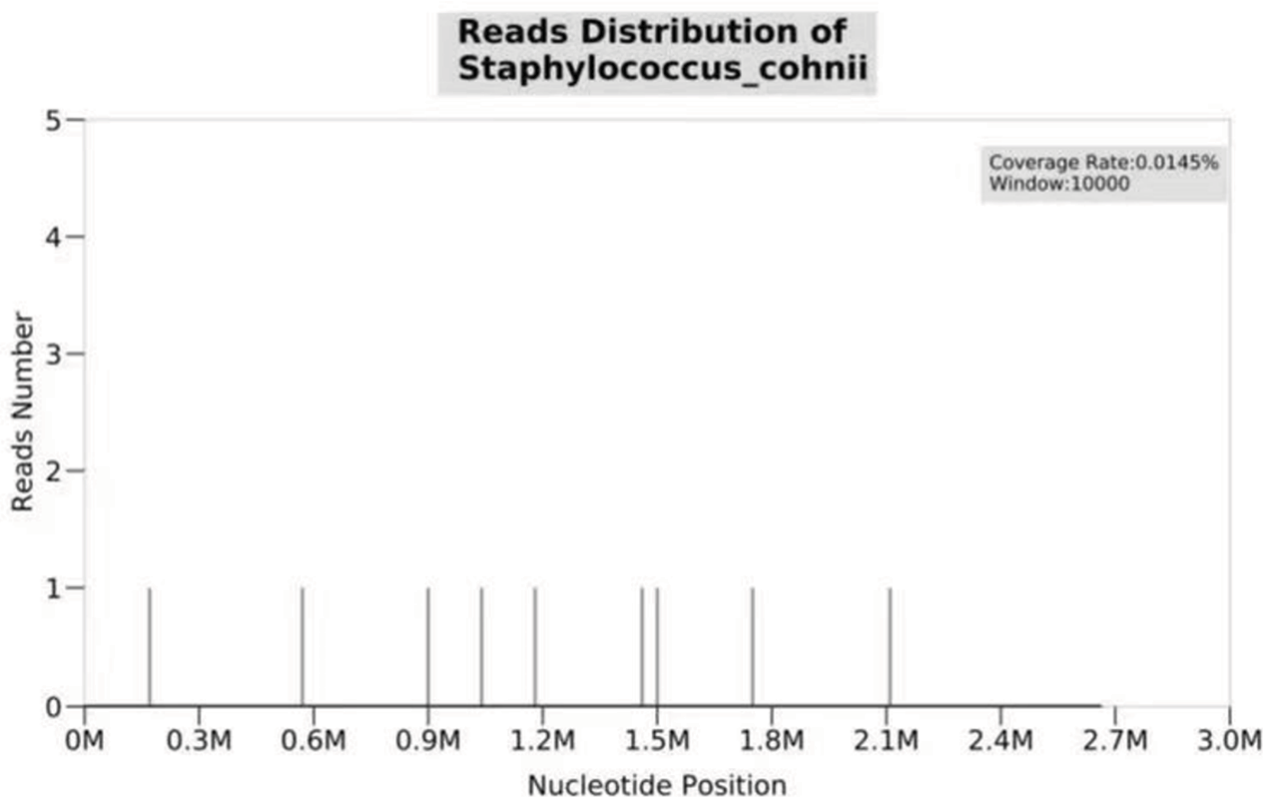
*S. cohnii* detected in ascites by mNGS.

Over more than one week of the empirical medication had little effect and the patient continued to complain of evident abdomen pain. Meanwhile, repeated ascites biochemistry, cytology, and bacterial culture tests were also performed, but the results remained negative in ascites bacterial culture. In order to determine the implicated organisms and to execute targeted treatment, the ascites samples were then sent for mNGS identification. DNA extraction, library construction, sequencing, data analysis and interpretation were performed by Beijing Genomics Institution (BGI). The mNGS method applied in the present case covers 12 classes of suspected pathogenic microorganisms, including 6350 bacteria, 1798 DNA viruses, 1064 fungi, and 234 parasites. The Fortunately, *Staphylococcus cohnii* (*S. cohnii*) was quickly detected by mNGS (**Figure 1**). The number of specific reads of *S. cohnii* was shown in **Table 1**, and the coverage of the identified genome calculated with the mapping of the detected reads was 0.0145%. Other bacteria, viruses, fungus and rickettsia were not detected by mNGS. According to the results, the antibiotics were immediately switched to linezolid (0.6g per 12h) and sulbactam/cefoperazone (4.5g per day).

**Table 1:** The mNGS result.

Bacteria screen result		
	Species	Reads count*
**1**	*Staphylococcus cohnii*	19
Fungus screen result		
	Species	Reads count*
**-**	–	–
Virus screen result		
	Species	Reads count*
**-**	–	–
Mycoplasma/Chlamydia/Rickettsia screen result		
	Species	Reads count*
**-**	–	–

*Reads count: sequences generated by high-throughput sequencing platform; -, no results detected.

**Figure 2:**
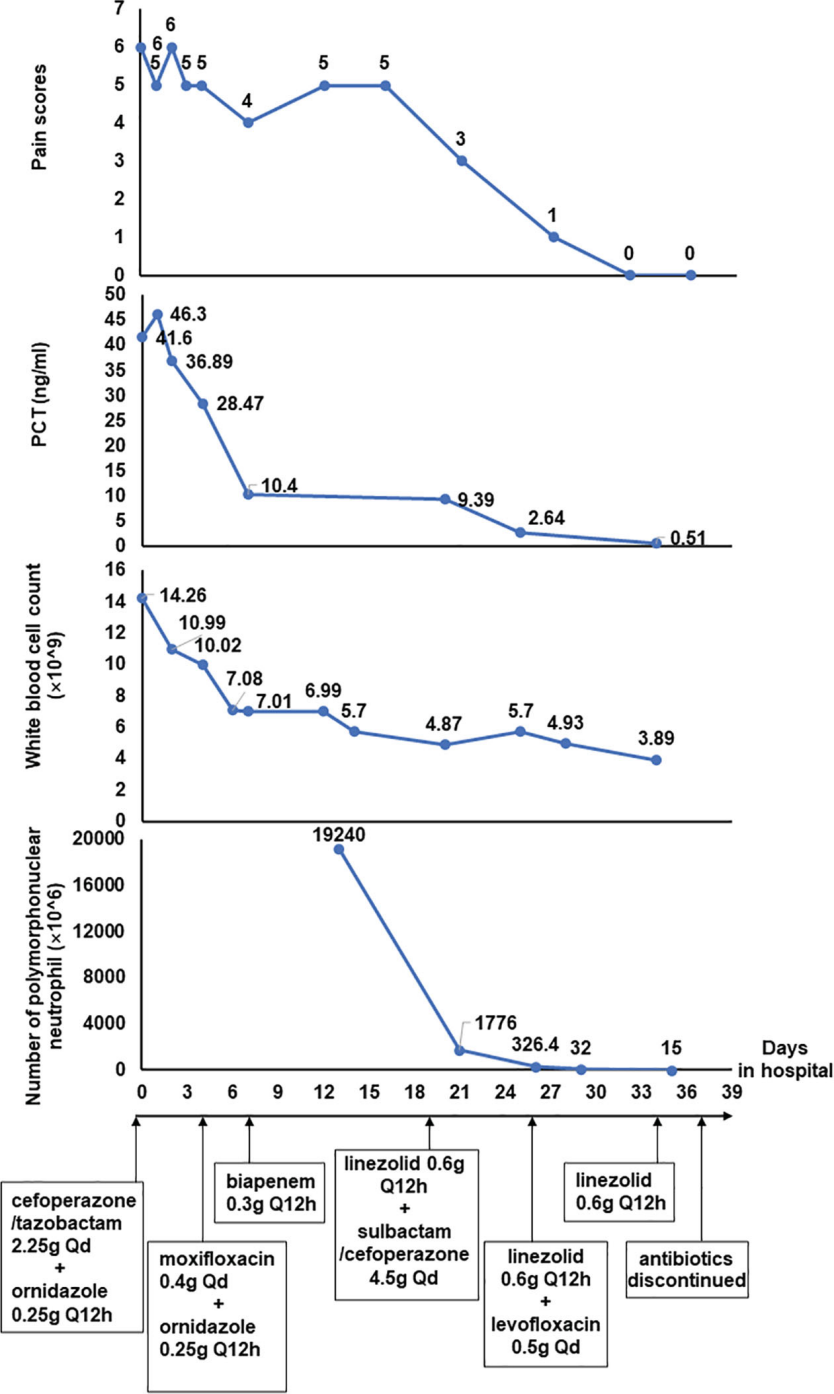
The laboratory dynamic monitoring indicators and treatment timeline of the patient.

Over a few days of treatment, the symptoms improved as the patient’s abdominal pain was significantly relieved, and there was also a noticeable decrease in the number of nucleated cells in ascites. Two weeks later, the laboratory investigation and ascites examination were repeated, and the results showed as follows: procalcitonin was 0.51ng/mL, the number of nucleated cells in ascites was 100×10^6/L and the Rivalta test was weakly positive. On day26 of hospitalization, the sulbactam/cefoperazone was changed to levofloxacin (0.5g per day). On day34 of hospitalization, the levofloxacin was discontinued, and the linezolid was discontinued on day37. The patient eventually recovered after 39 days of treatment in hospital and was discharged home from the hospital without obvious discomfort (**Figure 2**).

## 3 Discussion

SBP occurs in a considerable number of patients with cirrhosis and plays a vital role in the development of complications, progression to liver failure, as well as mortality. Bacterial ascites, which represent the first step in the development of SBP, were defined as polymorphonuclear neutrophil (PMN) count<C;0.25×10^9/L or positive culture results in the ascites fluid. Nevertheless, in the pathogenic identification of SBP, conventional microbiological tests may yield negative results due to prior antibiotic therapy or technical limitation. When conventional culture and serological testing failed to determine the likely infection etiology, mNGS examination of ascitic fluid specimens might play an important function. Some reports have demonstrated the advantages of mNGS application in diagnosis of infectious diseases, such as sepsis, meningitis, encephalitis, and pneumonia (Miao et al., 2018; Han et al., 2019; Peng et al., 2021; Piantadosi et al., 2021; Sun et al., 2022). However, as far as we know, there have been few reports on ascites, especially on SBP. The mNGS test not only provides a reliable basis for clinical precision medicine, but also facilitates a more rational antibiotic application, which in turn improves patients’ clinical prognosis.

In the present case, the patient went through a complex and tortuous process of treatment for over 1 month. With the initial empirical antibiotic treatment employed, the infectious parameters decreased, but there was no significant improvement in the patient’s symptoms of abdominal pain or imaging results. After the paracentesis, *S.cohni* was finally identified in the mNGS test of ascites. To our knowledge, it is the first report of the detection of *S. cohnii* in ascites by mNGS.

mNGS is a technique to obtain nucleic acid sequences in samples by rapid sequencing with the help of a second-generation sequencing platform, and further compare them with the genomic sequences of individual species to detect the species and proportion of microorganisms in the samples (Gu et al., 2019). Detection of pathogenic microorganisms using mNGS typically requires the following six steps, including collection of samples from the patient’s infection sites, extraction of nucleic acids, construction of a standard sequencing library, high-throughput sequencing, bioanalysis to identify pathogenic bacteria, and finally report interpretation (Simner et al., 2018). mNGS technology is rapidly moving from basic research to clinical laboratory. In addition, mNGS is changing the way physicians diagnose and treat infectious diseases with a wide range of applications, including antimicrobial resistance, human host gene expression transcriptomics, microbiome, and oncology, among others.

Though mNGS has unique advantages in clinical microbiology, its capability to detect certain difficult, undetectable, and low-burden microorganisms still needs to be improved. The type of extraction methods used by different laboratories also have an impact on mNGS results (Simner et al., 2018). The broad coverage of microorganisms brings a challenge to the interpretation of mNGS results. When interpreting results, technicians need to consider the pathogenicity and conditions of different microorganisms and set individualized reporting thresholds, while clinicians need extensive experience and specialized expertise (Han et al., 2019). In addition, the high cost of mNGS also prevents its widespread clinical application currently.

*S.cohnii* is an opportunistic pathogen that can cause urinary tract infections, bacteremia, and sepsis (Waldon et al., 2002; d’Azevedo et al., 2008; Soldera et al., 2013). Recently, *S.cohnii* endocarditis in the native valve was also reported in an 80-year-old patient, which indicated a poor prognosis (Motta et al., 2020). Once the treatment was switched to linezolid therapy, the patient recovered rapidly, suggesting *S.cohnii* infection may be the cause of SBP. The common pathogens of SBP are mainly gram-negative bacteria, such as *Escherichia coli*, *Klebsiella pneumoniae*, *Pseudomonas spp (* Dever and Sheikh, 2015; Fiore et al., 2019). However, *S.cohnii* infection of ascites has not been reported. In this case, the patient received regular hemodialysis and it might be a most likely risk factor, as the majority of the isolated organisms in ascites of patients with cirrhosis and dialysis were gram-positive (mainly *Staphylococcus*) *(* Nader et al., 2017). End-stage renal disease (ESRD) and hemodialysis expose patients to a higher risk of infection, especially in patients with comorbid other systemic diseases, such as liver cirrhosis, rheumatic diseases, which can lead to severe morbidity and mortality. Uremia in chronic renal insufficiency might account for immune dysfunction. Meanwhile, hemodialysis patients are at increased risk for bloodstream infections because the presence of vascular access creates possible entry points for pathogens (Nader et al., 2017; Aitkens et al., 2022).

In the present case, routine repeated ascites bacterial culture results were always negative. We considered that the sensitivity of conventional bacterial culture of ascitic samples was not strong. On the one hand, the empirical antibiotic treatment as soon as admission further reduced the positive rate of ascites culture. Furthermore, for typical bacterial cultures, many laboratories may define the detection thresholds in advance, however, this threshold might miss many potential infections. Through mNGS, we succeeded in rapidly detecting possible ascites infection pathogens that were not detected by ascites bacterial culture, demonstrating the speed and detectability of mNGS over conventional ascites bacterial culture. The number of pathogenic bacteria decreases with the use of antibiotics, while cultures appear to be falsely negative due to residual pathogens below the threshold for routine culture testing. Thus, the ascites mNGS test still detects bacteria after antibiotic administration, which is significantly stronger than conventional cultures. It is worth mentioning that Li, et al. have reported 3 cases using mNGS for the detection of infected ascites in cirrhosis, and they found that mNGS application showed limited effects in some settings (Li et al., 2022). Nevertheless, in another study, mNGS application played an important role in precision therapy in ascitic infection (Wu et al., 2023). The present case showed that the antimicrobial program guided by mNGS had a significant effect, demonstrating the promise of mNGS application in ascites and SBP.

## 4 Conclusion

In conclusion, we reported a case in which mNGS helped clinicians accurately identify S. *cohnii* infection in a patient with SBP. This case illustrated the potential application of mNGS in detecting pathogenic microorganisms in ascitic samples which were not detected by conventional culture and serological tests. It is believed that in the near future, with the cost of sequencing decreased, mNGS will be more and more widely used in the clinic to benefit more patients with SBP.

## Data availability statement

The datasets presented in this study can be found in online repositories. The names of the repository/repositories and accession number(s) can be found below: https://www.ncbi.nlm.nih.gov/, accession number, PRJNA1018443.

## Ethics statement

The studies involving humans were approved by Academic Committee of Tongji Hospital, Tongji Medical College, Huazhong University of Science and Technology. The studies were conducted in accordance with the local legislation and institutional requirements. The participants provided their written informed consent to participate in this study. Written informed consent was obtained from the individual(s) for the publication of any potentially identifiable images or data included in this article. Written informed consent was obtained from the participant/patient(s) for the publication of this case report.

## Author contributions

YL contributed to the manuscript preparation. QG and JL were responsible for the collection of clinical specimens and information. HH contributed to data analysis. PH contributed to the study design and review of the manuscript. All authors contributed to the article and approved the submitted version.
